# The Effects of Operational Conditions on the Respiration Rate of Tubificidae

**DOI:** 10.1371/journal.pone.0081219

**Published:** 2013-12-02

**Authors:** Juqing Lou, Yongqing Cao, Peide Sun, Ping Zheng

**Affiliations:** 1 Department of Environmental Engineering, Zhejiang University, Hangzhou, China; 2 School of Environmental Science and Engineering, Zhejiang Gongshang University, Hangzhou, China; Wageningen University, Netherlands

## Abstract

Tubificidae is often used in the wastewater treatment systems to minimize the sludge production because it can be fed on the activated sludge. The process conditions have effect on the growth, reproduction, and sludge reduction efficiency of Tubificidae. The effects of the water quality, density of worms, pH, temperature and dissolved oxygen (DO) concentration on the respiration rate of Tubificidae were investigated to determine the optimal conditions for the growth and metabolism of the worms and reveal the mechanisms involving the efficient sludge reduction in terms of these conditions. It was observed that the respiration rate was highest in the water discharged from an ecosystem that included symbiotic Tubificidae and microbes and was lowest in distilled water. Considering density of the worms, the highest rate was 81.72±5.12 mg O_2_/g(dry weight)·h·L with 0.25 g (wet weight) of worms in 1 L test flask. The maximum Tubificidae respiration rate was observed at a pH of 8.0±0.05, a rate that was more than twice as high as those observed at other pH values. The respiration rate increased in the temperature range of ∼8°C–22°C, whereas the rate declined in the temperature range of ∼22°C–30°C. The respiration rate of Tubificidae was very high for DO range of ∼3.5–4.5 mg/L, and the rates were relatively low for out of this DO range. The results of this study revealed the process conditions which influenced the growth, and reproduction of Tubificidae and sludge reduction at a microscopic level, which could be a theoretical basis for the cultivation and application of Tubificidae in wastewater treatment plants.

## Introduction

Aerobic wastewater treatment plants (WWTPs) produce large quantities of sludge containing various pollutants like heavy metals, pathogens and micropollutants. The estimated cost of treatment and disposal of waste sludge in WWTPs represents half, or even as much as 60% of the total cost of wastewater treatment [1],[2]. Currently, conventional sludge reduction methods, including mechanical, physical, chemical, electrical and biological methods, are in use [3]; however, these methods are energy and cost intensive [4], [5]. In view of stricter regulations, new technologies should be developed to reduce the production and disposal of waste sludge. Biological wastewater treatment involves several techniques for processing waste sludge, such as lysis-cryptic growth [6], metabolic uncoupling [1], [7] and maintenance metabolism and predation on bacteria [7], [8]. Compared with other strategies, the predation method has attracted recently considerable attention because of several obvious advantages such as energy saving and no secondary pollution production [9], [10]. One of the typical predation methods is the application of aquatic worms for sludge reduction [11], [12], which would decrease the amount and volume of excess sludge by metabolic processes. The volume of sludge is reduced after treatment because the sludge flocs are compacted, and meanwhile, the sludge amount is distinctly decreased [13],[14], because worms eat sludge as food per day several times the their own weight[9]. So, a large amount of sludge is consumed by the worms, and the amount of new worm biomass and worm feces are much lower than the sludge consumed. Indeed, this method may be the most cost-effective and environmentally friendly for sludge reduction in WWTPs [15], [16]. Several worm species present specific characteristics that make them suitable for such applications. Oligochaetes may represent 50–60% and even up to 100% of the benthic biomass [3] and are the largest type of metazoa observed in WWTPs [17], [18]. The principal types of worms found in activated sludge systems are Naididae (such as Tubificidae) [2] and, Aeolosomatidae [8], [9]. The two most common species, *Tubifex tubifex* and *Limnodrilus hoffmeisteri* Clap., are reported to survive in polluted water environment [19], [20] or in highly polluted, oxygen-poor and/or toxic ecosystems [21], [22]. These species being the indicators of organic pollution are capable of growth and stable sludge reduction. Also, they have shown good survival and reproductive rates in activated sludge during wastewater treatment processes [13], [19], [23]. For these reasons, Tubificidae were selected for this study.

The sludge reduction by worms was closely related to the process conditions in applications involving aquatic worms. Some researchers [3], [4], [13] have already reported that the conditions, such as temperature, pH, dissolved oxygen (DO) etc had a clear effect on the worms eating waste sludge. So, high sludge reduction efficiency could only be achieved through stable growth and good reproduction of worms in WWTPs. However, growth, reproduction, and survival of aquatic worms are inevitably influenced by the conditions of aquaculture systems, e.g., temperature and pH [24], [25]. Additionally, the respiration rate is an indicator of metabolism and growth of Tubificidae, which is also affected by these conditions. Therefore, it is necessary to investigate the effects of conditions on the respiration rate, the growth of the worms, and the sludge reduction capability of worms. Although the effects of such pollutants as cadmium and sodium pentachlorophenate on the respiration of tubificid worms [26]–[29] have been addressed, however, relationship between changes in the respiration rate and natural conditions in the WWTPs environment has never been investigated.

Therefore, this study investigates comprehensively the effects of the water quality, worm density, pH, temperature and DO concentration on the respiration rate of tubificid worms. The results of the study revealed a suitable sludge reduction habitat for Tubificidae and gave insights into the conditions related to sludge reduction efficiency. The results of the study could offer rational explanation for how worms' growth and sludge reduction efficiency were affected by the process conditions, and how application of aquatic worms in sludge reduction can be improved.

## Materials and Methods

### Materials

#### Tubificid worms

Tubificidae (primarily *T. tubifex*, with some *L. hoffmeisteri*) specimens were collected from the Shengxin Aquarium, Lanling flower and bird market, Shanghai. The worms to be tested were separated from other organisms using standard graduated sieves and materials. They were kept at approximately 20°C in the laboratory under slow stream of water. Single worm was selected with a medicine dropper after repeated washing with the help of tap water to minimize possible injury. The worms selected were of fairly uniform appearance and size (∼6–7 cm), red in color, had no apparent injuries and responded rapidly (the posterior or anterior end of a worm rapidly withdraws and coil their body) to physical stimuli [30], [31]. The animals were not fed during the experiment. The respiration rate tests were performed with discharged water unless the experiment involved differences in the water type.

Test solutions. The experiments were performed in tap water, distilled water and water discharged from system consisting of symbiotic Tubificidae and microbes. The symbiotic system was operated as a sequencing batch reactor (SBR), in which the mixed-liquor suspended solids (MLSS) was about 3500 mg/L, pH ∼7–8, temperature was at 25±1°C, the DO concentration was about 3 mg/L, the Chemical Oxygen Demand (COD) was about 50 mg/L and ammonia concentration of discharged water was about 7.2 mg/L.

The discharged water was autoclaved before use to eliminate the effect of microorganisms. The pH of the solutions was adjusted with 1.0 mol/L HCl or 1.0 mol/L NaOH.

### Methods

#### Dry weight and wet weight of worms

The worms were placed on perforated aluminum foil kept over absorbent paper to measure the wet weight (ww) and gentle pressure was applied to the aluminum foil paper to allow the underlying paper to absorb the water from the surface of the worms. The worms were weighed using an analytical balance after drying [14].

Then the worms were heated overnight at ∼103–105°C for a constant weight in order to measure their dry weight [4], [32].

#### The effect of water quality on the respiration rate

Worms samples of 0.25 g (ww) were placed in tap water, distilled water and discharged water from the symbiotic ecosystem. All the tests were performed in triplicate at a temperature of 22±1°C, a pH of 8.0±0.05 and a DO concentration of ∼3.5–4.0 mg/L. The original characteristics of distilled water, tap water and discharged water are given in Table 1.

**Table 1:** The original characteristics of distilled water, tap water and discharged water.

	pH	DO (mg/L)	COD (mg/L)	hardness^a^ (mg/L)
distilled water	6.07	7.24	0	0
tap water	7.20	6.08	2.11	87.9
discharged water	7.78	2.05	49.2	77.2

a The hardness of water was represented as CaCO_3_, mg/L.

#### The effect of worm density on respiration rate

Worms samples of 0.25 g, 0.50 g, 0.75 g, 1.00 g and 1.50±0.01 g (ww) were added to the 1 L (standard sample volume) discharged water to determine the effect of worm density on the respiration rate. The pH, temperature and DO were controlled at 8.0±0.05, 22±1°C and ∼3.5–4.0 mg/L, respectively.

#### The effect of pH on respiration rate

The tests were conducted at four different pH values of 6.0, 7.0, 8.0, and 9.0 to investigate the effect of pH on the respiration rate. Each test was performed in triplicate. A Tubificidae sample of 0.25 g (ww) was added to a flask with discharged water. The temperature was controlled at 22±1°C for all experiments, and pH was controlled at the pre-designated set point ±0.05 with the help of NaOH (1.0 mol/L) and HCl (1.0 mol/L).

#### The effect of temperature on respiration rate

A Worms sample of 0.25 g (ww) was tested individually at six different temperatures 8°C, 15°C, 22°C, 25°C, 27°C and 30°C to investigate the effect of temperature on the respiration rate. All the experiments were performed in discharged water at a pH of 8.0±0.05 and a DO concentration of ∼3.5–4.0 mg/L.

Each test was performed in triplicate. The temperature was controlled by the water bath of BI-2000 electrolytic respirometer.

#### The effect of DO concentration on respiration rate

The DO concentrations were controlled with air and/or nitrogen gas diffusers [4] at 1.5±0.2 mg/L, 2.5±0.2 mg/L, 3.5±0.2 mg/L, 4.5±0.2 mg/L, 5.5±0.2 mg/L, and 6.5±0.2 mg/L. A Tubificidae sample of 0.25 g (ww) was tested in discharged water at a pH of 8.0±0.05 and a temperature of 22±1°C.

#### Determination of respiration rates

**Figure 1:**
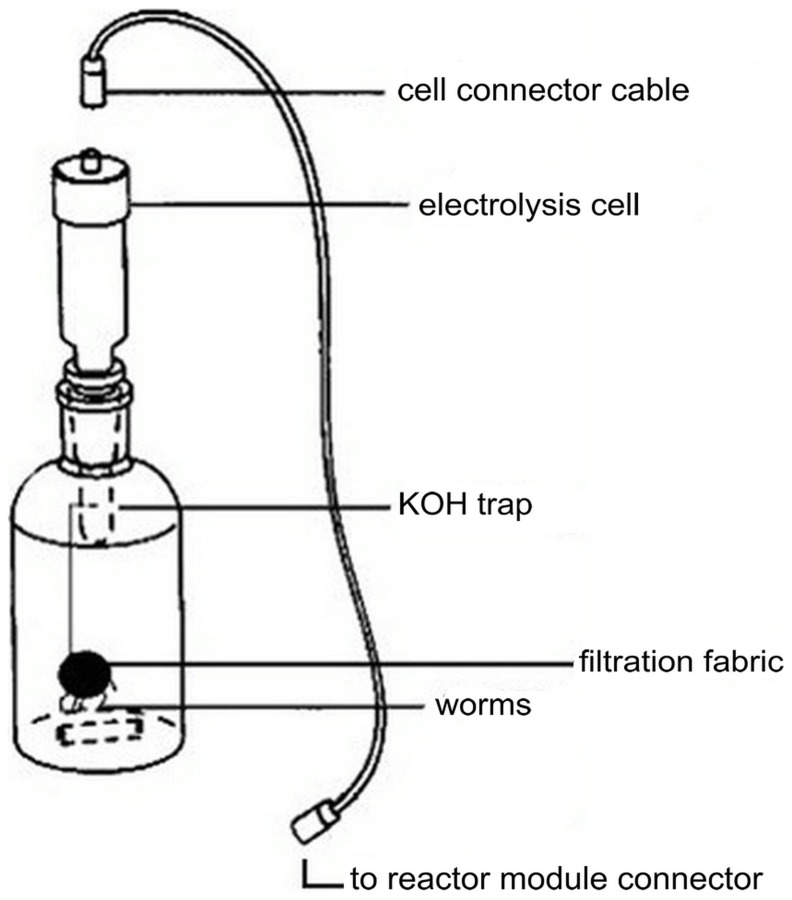
The apparatus used to measure respiration rates.

An electrolytic respirometer was often used to monitor oxygen consumption by microorganism [33], [34]. The BI-2000 electrolytic respirometer (Bioscience Inc., Bethlehem, PA) is shown in Figure 1 [35]. The flasks (total volume is 1225 mL, and standard sample volume is 1000 mL) were shaken continuously in a water bath at a constant temperature of 22°C, with the exception of the tests involving temperature effects. Carbon dioxide in the apparatus was absorbed by filter paper strips placed in a KOH trap and saturated with 45% KOH to form a slight vacuum in the reactor (relative to the external atmospheric pressure), and this slight pressure difference was detected by pressure sensor. The electrolysis cell dissociated dilute sulfuric acid to produce oxygen, in an amount proportional to the current, which was added to the reactor until the pressure returned to equilibrium. The hydrogen generated by this reaction was exhausted through a vent. The worms were wrapped in filter cloth and hung in the reactor during the test, which was not initiated until the water bath reached the desired temperature. The respiration rate was expressed as mg/L O_2_ consumed per gram (dw) of worms per hour, and it was presented as mean ± SD (standard deviation).

#### Other item analysis

The dry weight (dw) and wet weight (ww) of Tubificid worms were determined using an EL104 analytical balance. The DO concentration was measured by a DO meter (FE20 Meter Toledo), and pH was measured by Tengine EDO pH meter. The COD and hardness of water were measured according to standard methods (APHA, 1999) [36].

### Ethics Statement

The owners of Shengxin Aquarium have permitted to use these worms in this study, and animal procedures were performed according to laboratory animals use guidelines which were approved by the Ethics Committee of Zhejiang Gongshang University, Hangzhou, China.

## Results and Discussion

### Ratio of dry weight to wet weight of Tubificid worms

**Figure 2:**
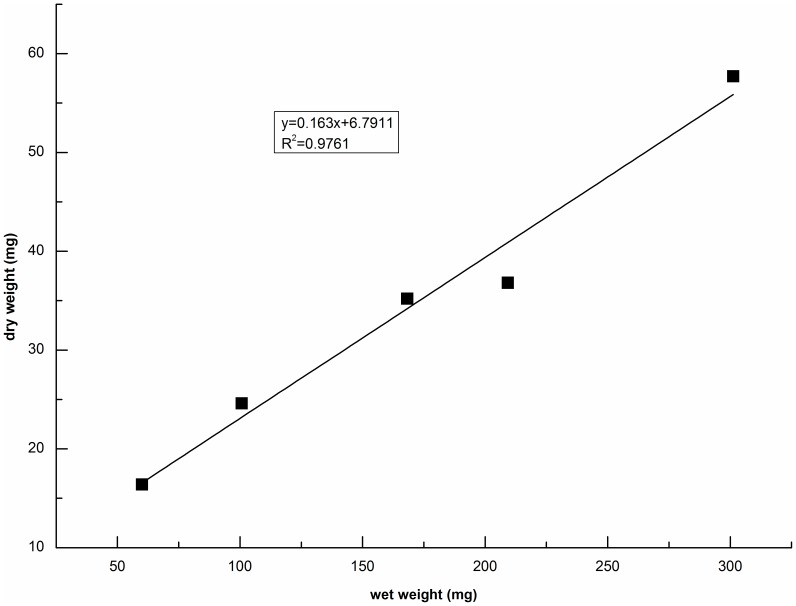
Dry weight vs. wet weight of Tubificidae worms.

The dry weight to wet weight ratio of worms was approximately 16.3% (Figure 2), which was quite close to 0.161±0.015 which has previously been reported for *T. tubifex* [37]. Elissen[13] determined dry weight to wet weight ratio for different types of aquatic worms: 17% for sessile Tubificidae and 13% for *L. variegatus*. The value found by Elissen for Tubificidae differed slightly from that found in the present study, perhaps due to differences in the length, diameter and biovolume of the worms used in two studies [13], [38].

### The effect of water quality on respiration rate

**Figure 3:**
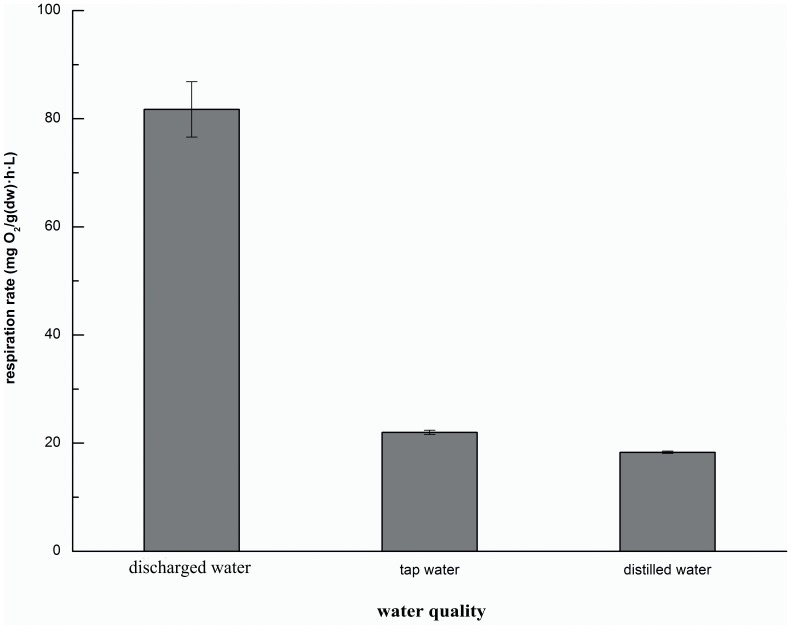
Respiration rates in water with different qualities (n = 3, error bars  =  SD).

The results of the experiment with different water sources are shown in Figure 3. The highest respiration rate was found in the water discharged from the symbiotic ecosystem, which was 81.72±5.12 mg O_2_/(g(dw)·h·L), compared to 21.99±0.39 and 18.31±0.22 mg O_2_/(g(dw)·h·L) in tap water and distilled water, respectively. These findings showed that the operational sludge reduction system might be favorable for the growth and metabolism of Tubificidae. Distilled water could be considered for Tubificidae growth as a pure substance without any additional components, in the absence of trace elements. Consistently, the respiration rate in tap water was slightly higher than that in distilled water. Moreover, discharged water contained many other micronutrients energizing microbial growth and metabolism, which could be one of the reasons why aquatic worms could grow well in WWTPs, and achieve high sludge reduction efficiency.

### The effect of worm density on respiration rate

**Figure 4:**
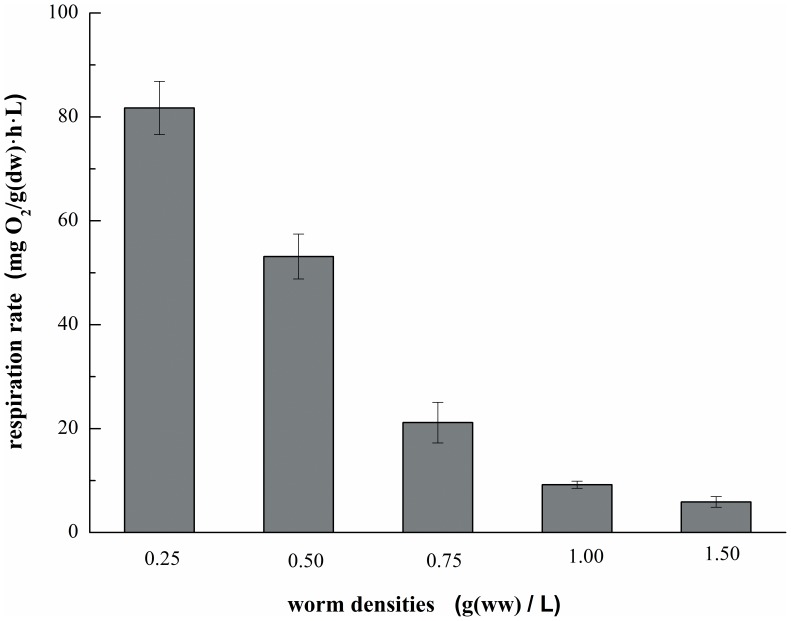
Respiration rates for different worm densities (n = 3, error bars  =  SD).

Figure 4 shows that the worm density had a significant effect on the respiration rate. The respiration rates found with 0.25, 0.50, 0.75, 1.00 and 1.50 g (ww)/L of worms were 81.72±5.12, 53.13±4.34, 21.17±3.90, 9.20±0.69 and 5.89±1.04 mg O_2_/(g(dw)·h·L), respectively. It was likely that the markedly decreased respiration rate with an increasing density of worms was a result of the tendency of the worms forming a structure termed “ball cluster”, a typical behavior of Tubificidae. Indeed, the increased density of worms resulted in the formation of a larger ball, leading to oxygen transmission and the respiration of the worms was hindered in the middle of the ball. This effect has been reported by previous investigators [39], [40], who found that less oxygen was consumed by the animals if they formed a ball. Wei et al.[41] have even reported that the quality of the effluent could be worse when the worm density was higher. The density of inoculated worms should be maintained within an appropriate range in the practical application.

### The effect of pH on respiration rate

**Figure 5:**
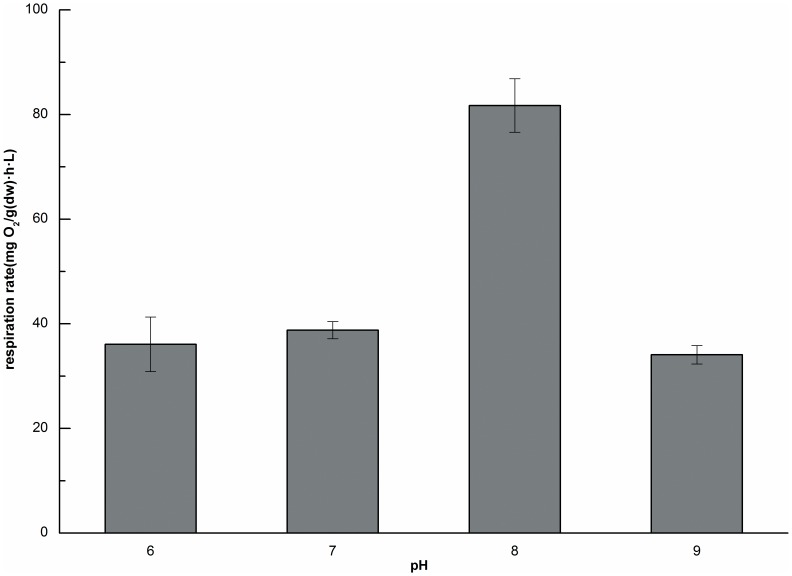
Respiration rates at different pH values (n = 3, error bars  =  SD).

Several pH values were investigated experimentally to determine the possible effect of pH on the respiration rate. The respiration rate increased as the pH changed from 6.0 to 8.0 as shown in Figure 5, and reached a maximum rate at pH 8.0, and then decreased when pH increased further. Although the respiration rate in the range of ∼6.0–8.0 was slightly higher than that at pH 9.0. A change in pH from 7.0 to 6.0 did not alter the respiration rate significantly, which was in accordance with the research of Fowler [28], there was no difference in the oxygen consumption rate when pH shifted from 7.6 to 6.1. These findings indicated that neutral pH or pH values approaching the alkaline range were beneficial for Tubificidae. The environment under these pH values range is suitable for most of microorganisms, such as nitrifying and denitrifying bacteria [42], [43]. The pH in a domestic sewage treatment plant is generally ∼6–8 [44], a range that could be benefiting the application of worms and helpful for improving the sludge reduction efficiency.

### The effect of temperature on respiration rate

**Figure 6:**
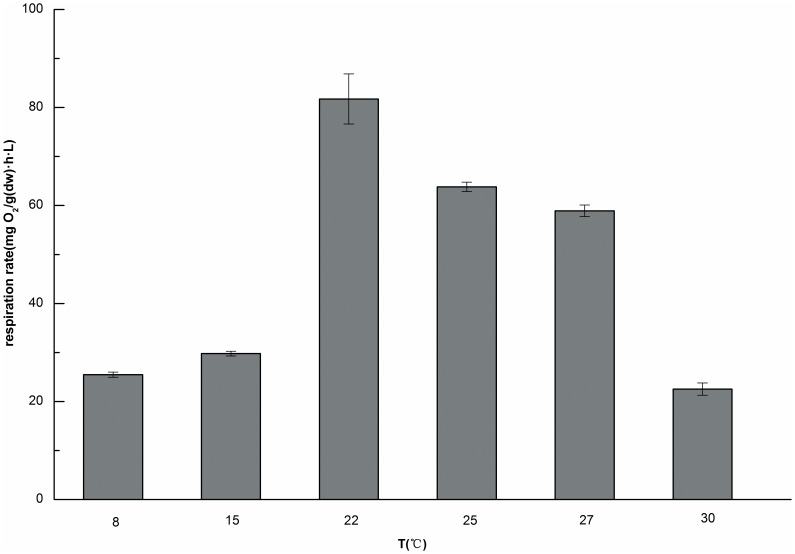
Respiration rates at different temperatures (n = 3, error bars  =  SD).

Temperature is an important factor for aquatic organisms. For example, temperature influences the respiration[28], growth and reproduction of Tubificidae [23], [45], [46]. As revealed in this study, the Tubificidae specimens studied were found to be very sensitive to temperature, as shown in Figure 6. The respiration rate increased from 25.46±0.52 mg O_2_/(g(dw)·h·L) to 81.72±5.12 mg O_2_/(g(dw)·h·L) as the temperature increased from 8°C to 22°C. The respiration rate was maximum at approximately 22°C and then the rate decreased with further increase of temperature. The rate remained relatively high when temperatures varied from ∼22–27°C but was considerably low below 22°C. The oxygen consumption rate and activity level decreased significantly when temperature dropped from 25°C to 0°C according to available report [28]. The aggregation of the worms became detached at a temperature of 30°C. The available literature [4] showed that temperature clearly affected both the sludge digestion efficiency and sludge reduction by Lumbriculus variegatus. The optimum consumption rate by the worms occurred at approximately 15°C, whereas the maximum decrease in TSS occurred at 10°C. A gradual decrease in the sludge digestion efficiency occurred when the temperature increased from 10 to 20°C. In contrast, Guo et al.[47] reported that the digestion efficiencies increased as the temperature increased from 15°C to 25°C and that the maximum digestion efficiencies of TSS and VSS occurred at 25°C. Although it is evident from these findings that temperature is an important factor influencing sludge reduction by worms. Because Tubificidae are ectotherms, their metabolism and physiological state [48] are directly dependent upon the water temperature, with a greater metabolic rate at higher temperatures and reduction in the animals' activity and metabolic rate at lower temperatures. But the optimal temperature value remains unclear because a unified conclusion could not be drawn from the available literature. Some researchers think that optimal temperature for growth for Tubificidae usually is in the range of ∼20–25°C [3], [46], but others think that the optimum temperature is 25°C[3], [45]. Reynoldson[49] reported that growth of T. tubifex and Limnodrilus hoffmeisteri (Tubifieidae, Oligoehaeta) occurred only over a very similar narrow range at ∼10–13°C. So, we could not specify a precise optimum temperature. However, the results of the current study could suggest that the optimal temperature for Tubificidae is at approximately 22°C, a finding that is consistent with the literature [46], [50]. In addition, the temperature varies with the season and climate. However, the temperature of WWTPs is relatively stable, and the range in a typical WWTP is ∼20–24°C [51], which is beneficial for sludge reduction by worms. So a temperature greater than 30°C should be avoided and temperature range of ∼20–27°C should be maintained in sludge reduction system involving worms.

### The effect of DO concentration on respiration rate

**Figure 7:**
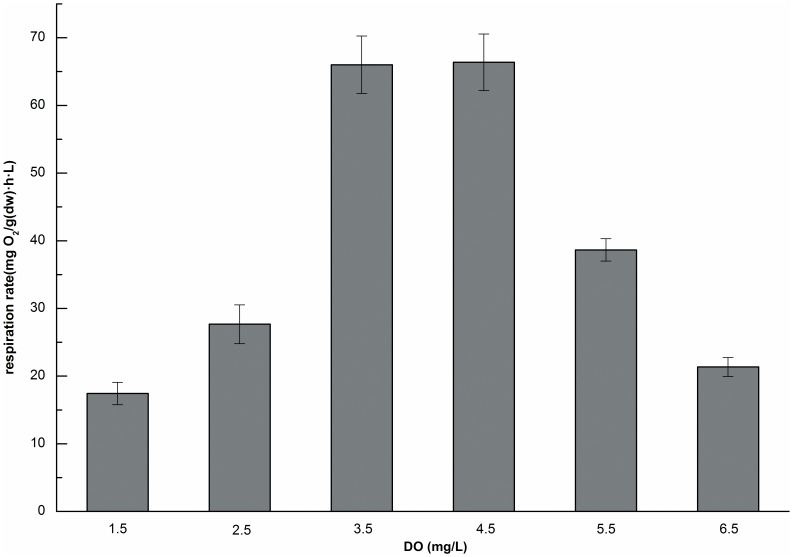
Respiration rates at different DO concentrations (n = 3, error bars  =  SD).

DO is important parameter for the growth and reproduction of aquatic worms, for example, it can affect bioturbation by infaunal burrowers [52], and it has also been reported to affect both the sludge reduction efficiency and consumption rate [4]. As shown in Figure 7, changes in the DO concentrations represented an environmental stress for Tubificidae. The respiration rate increased when the DO concentrations increased from 1.5 mg/L to 6.5 mg/L and then the rate decreased gradually. The respiration rate reached its maximum value when DO concentration was at 4.5 mg/L, which was 3.8 and 3.1 times higher than that observed at the lowest and highest concentrations, respectively. In this study, DO concentrations between 3.5 and 4.5 mg/L were found to benefit Tubificidae. However, a high oxygen input represented an increase in energy input, and a strong disturbance in the flow as a result of excessively high agitation might produce a low sludge reduction rate [53]. Fowler [28] has shown that the degree of vigor of undulation and elongation varied directly with the decrease in oxygen pressure. Under low DO condition, worms would extend their bodies and squeeze into each other to compete fiercely for more oxygen, so that they were washed away easily [54]. Also, Tubificidae showed weight loss under anoxic conditions [44], [49], and the Tubificidae reduction efficiencies of TSS and VSS were negative in a DO concentration range of ∼0–0.5 mg/L [47]. Clearly, low and high DO concentrations should be avoided. So, an intermediate DO concentration should be identified for practical use which involves both economical benefits and high respiration rate of the worms. In actual installations, the DO level was maintained at approximately 4 mg/L in an aerobic tank, in which worms had a suitable respiration rate and also survive and reproduce well [44].

## Conclusions

The respiration rates of Tubificidae in distilled water, tap water and water discharged from the system consisting of symbiotic Tubificidae and microbes were investigated. The highest respiration rate was achieved with the discharged water (i.e., 81.72±5.12 mg O_2_/(g(dw)·h·L)), followed by tap water (i.e., 21.99±0.39 mg O_2_/(g(dw)·h·L)), and the lowest rate was found with the distilled water (i.e., 18.31±0.22 mg O_2_/(g(dw)·h·L)).

The density of worms had a great effect on the respiration rate, which decreased dramatically with the increase in the density of worms. The highest respiration rate was found at a worm density of 0.25 mg (ww)/L, with a maximum value of 81.72±5.12 mg O_2_/(g(dw)·h·L). Thus, it is necessary to maintain an appropriate density of worms in the sludge reduction system.

A high respiration rate was observed in the temperature range of ∼20–27°C, and the highest rate was three times higher than the rate at 8°C. These results indicated that an optimal temperature of 22°C was beneficial for the survival and metabolism of Tubificidae.

Observations at different pH values showed that the highest respiration rate occurred at a pH of 8.0. Differences among the respiration rates at other pH values were markedly less with the rates at other pH values less than half of the rate observed at pH 8.0.

The respiration rate of the worms was strongly influenced by the DO concentration. The highest rate occurred within an optimum DO range of ∼3.5–4.5 mg/L. The rate first increased and then decreased as DO increased from 1.5 to 6.5 mg/L. Excessively high or low DO concentrations were not favorable for the worms.
